# Indigestion and Its Management

**Published:** 1873-01

**Authors:** 


### ARTICLE VI.

### *Indigestion and its Management.*

Dr. Bradford S. Thompson, in a paper read before the Medical Library and Journal Association of New York, says the causes of dyspepsia are two-fold: 1st. Such as act directly upon the stomach. 2nd. Such as act through the medium of the general system. Among the first may be mentioned eating and drinking certain articles of food. The immoderate use of opium and tobacco, in all their forms, often produce it. Those causes which operate through the medium of the general system are as follows: intense study, attention to business within doors, excessive venery, grief, and exposure to cold. In detailing the best mode of treatment, the first indication, when acute indigestion presents itself from the presence of completely insoluble vegetable fiber, is to evacuate the contents of the stomach by emetics. By this procedure that viscus is not only relieved from noxious substances, but the organization is prepared for the operation of other remedies. As co-operating in the same design are laxatives, but with regard to the selection, some discrimination is requisite. All the salines, as well as the drastic purgatives, are to be avoided. The only exception is rhubarb, which in very many cases may be advantageously administered. Indeed, it seems to be particulary adapted to cases of this character by its tonic property. The only objection to this remedy is that it is apt to leave behind a tendency to constipation, which, however, is obviated by the combination of calcined magnesia, or bi-carbonate of soda. The condition of the alimentary canal being rectified, tonics are then resorted to. Many of the vegetable bitters are very useful, particularly gentian, quassia, hop and columbo. Of all tonics, the hop, or its active principle, lupulin, is perhaps, decidedly the best when dyspepsia arises from drunkenness or debauchery. Dr. James R. Woods' formula is to be recommended in the latter cases:

℞ Tinct. Valerian Aromat., - - - -
Tinct, Lupulin, - - - - aa ℥ ij.
Tinct. Cardamom, - - - - ℥ ss.
M. S. A teaspoonful as required.

In ordinary cases the mineral tonics are preferable. The carbonate of iron in ten grain doses, with a small proportion of the fluid extract of ginger, may be prescribed with utility, but the sulphate of iron, after repeated experience, is the most efficacious of the chalybeates, and should be administered to the patient in the form of pills according to the subjoined formula:

℞ Ferri. Sulph., - - - - - ʒ j.

Acaciæ Arabicæ, - - - - grs. xxx.

M.

This to be made into thirty pills, one of which may be administered three or four times in twenty-four hours. For heart-burn, emetics are to be avoided, and give lime water mild or any of the alkalines. In gastrodynia McMunn's elixir of opium, creasote or carbolic acid are serviceable. In the treatment of pyrosis, or water brash, antacids are to be resorted to, particularly the saccharated solution of lime water. Indigestion is often exceedingly intractable, and sometimes will not yield to ordinary remedies. It often appears to be riveted and established by long continued habit; to meet this indication nothing is so efficacious as calomel in small doses.

Dr. Chambers' favorite tonic in chronic indigestion is quinine in two-grain doses, in lemon juice sufficient to dissolve it, and dilute to convenient bulk. Its action seems to be principally on the mucous membrane of the mouth, œsophagus and stomach, which it astringes and tones up to a healthy state, restraining the secretion of mucous, and making its special secretions more active. To quinine he adds from one-twenty-fourth to one twentieth of a grain of hydrochlorate of strychnia. In children, Bodault's wine of pepsin is highly recommended to meet certain indications.

In regulating the diet, impress upon the patient the necessity of observing the subjoined rules: 1st. Enjoin frequent and regular eating in the majority of cases; 2nd. Let the diet be simple, consisting always of one article; 3d. Drink little or nothing while eating; 4th. Exercise should not be permitted directly after eating. Beer, port wine and undiluted liquors should never be given, as they almost always sour on the stomach and disagree with the patient. —*American Practitioner.*

---

